# A French National Survey on Clotting Disorders in Mastocytosis

**DOI:** 10.1097/MD.0000000000001414

**Published:** 2015-10-09

**Authors:** Ana B. Carvalhosa, Achille Aouba, Gandhi Damaj, Danielle Canioni, Chantal Brouzes, Emmanuel Gyan, Stéphane Durupt, Isabelle Durieu, Pascal Cathebras, Nathalie Costédoat-Chalumeau, David Launay, Benoit Pilmis, Stephane Barete, Laurent Frenzel, Olivier Lortholary, Olivier Hermine, Cedric Hermans, Marie-Olivia Chandesris

**Affiliations:** From the French Reference Centre for Mastocytosis (CEREMAST), Necker Children's Hospital, Assistance Publique - Hôpitaux de Paris (APHP), France (ABC, GD, DC, CB, SB, LF, OL, OH, M-OC); Haemostasis and Thrombosis Unit, Haemophilia Clinic, Division of Haematology, St-Luc University Hospital, Brussels, Belgium (ABC, CH); Department of Internal Medicine, Caen University Hospital, France (AA); Department of Haematology, Caen University Hospital, France (GD); Department of Pathology, Necker Children's Hospital, APHP, France (DC); Department of Biological Haematology, Necker Children's Hospital, APHP, France (CB); Department of Haematology and Cell Therapy, Tours University Hospital, France (EG); Department of Internal and Vascular Medicine, Lyon Sud University Hospital, France (SD, ID); Department of Internal Medicine, Saint-Etienne University Hospital, France (PC); Department of Internal Medicine, Cochin Hospital, APHP, France (NC-C); Department of Internal Medicine, Lille University Hospital, France (DL); Infectious Diseases Department, Necker Children's Hospital, APHP, France (BP, OL); Department of Dermatology, Tenon University Hospital, APHP, France (SB); Department of Haematology, Necker Children's Hospital, APHP, France (LF, OH, M-OC); and Sorbonne Paris Cité, Paris Descartes University, Imagine Institute, Paris, France (LF, OL, OH, M-OC).

## Abstract

Mastocytosis is characterized by a clonal mast cell proliferation with organ infiltration and uncontrolled degranulation. Although not characteristic and poorly explained, some patients develop clotting abnormalities.

We retrospectively identified patients with established diagnosis of mastocytosis and related clotting abnormalities (clinical and/or biological) using the national French Reference Centre for Mastocytosis database.

From our cohort of 14 adult patients with clotting abnormalities (median age 46 years [range 26–75]), 4 had a presentation suggestive of a primary hemostasis disorder alone (by their symptoms and/or abnormal clotting tests [PFA, von Willebrand's disease [vWD] screening]) and 10 had a laboratory impairment of secondary hemostasis. Among these, 7 had bleeds characteristic of a coagulation cascade disorder (severe/life-threatening in 5 and mild in 2 patients). Clotting abnormalities were of variable severity, typically related to intense crisis of degranulation, such as anaphylactic reactions, and/or to severe organ infiltration by mast cells. Importantly, classical hemostatic management with platelet transfusion, fresh frozen plasma, or vitamin K infusions was unsuccessful, as opposed to the use of agents inhibiting mast cell activity, particularly steroids. This illustrates the crucial role of mast cell mediators such as tryptase and heparin, which interfere both with primary (mainly via inhibition of von Willebrand factor) and secondary hemostasis. There was interestingly an unusually high number of aggressive mastocytosis (particularly mast cell leukemia) and increased mortality in the group with secondary hemostasis disorders (n = 5, 36% of the whole cohort).

Mast cell degranulation and/or high tumoral burden induce both specific biologic antiaggregant and anticoagulant states with a wide clinical spectrum ranging from asymptomatic to life-threatening bleeds. Hemostatic control is achieved by mast cell inhibitors such as steroids.

## INTRODUCTION

Mastocytosis is a rare clonal hematological disorder characterized by (1) the proliferation and accumulation of neoplastic mast cells in ≥1 organs (the skin and the bone marrow being predominantly involved) and (2) their unregulated activation/degranulation (ie mast cell mediators release), resulting in mast cell activation symptoms/syndrome (MCAS).^1^ The classification of mastocytosis is based on signs of organ infiltration,^2^ with or without organ enlargement (B-findings) and dysfunction (C-findings). It distinguishes localized mastocytosis (cutaneous mastocytosis [CM]) from systemic mastocytosis (SM). Systemic mastocytosis can be indolent (ISM; no B- nor C-findings), smoldering (SSM; ≥2 B-findings and no C-findings), aggressive (ASM; ≥1 C-finding), and associated with a clonal hematological nonmast cell lineage disease (SM-AHNMD). With regard to prevalence, 880 patients are regularly followed-up at the national French Reference Centre (CEREMAST) located at Necker Children's Hospital. We estimate a total of ∼2000 cases of mastocytosis in France with 80% CM or ISM, 15–20% ASM or SM-AHNMD, <1% MCL, and an unknown prevalence of SSM (CEREMAST, unpublished data).

In terms of prognosis, in indolent forms life expectancy is similar to that of the general population but MCAS remains a cause of an all too often underestimated burden and disability for patients.^3^ In ASM and SM-AHNMD, life expectancy is significantly reduced with a respective median survival of 6.7 years and 4.4 years, depending on organ dysfunction as well as AHNMD type and progression.^2,4^ Finally, mast-cell leukemia (MCL) usually presents with rapid organ failure as well as florid MCAS, including anaphylactic shock and is still associated with a catastrophic median survival of around 6 months.^2,5^

In addition to classical symptoms of mast cell degranulation and organ infiltration, clotting disorders have been reported in 13 patients to date, ranging from simple ecchymosis tendency to severe/life-threatening bleeds (epidural hematoma, intracerebral hemorrhage, severe gastrointestinal bleeding, massive postsurgical blood loss, hemorrhagic pleural effusion requiring ventilation).^6–18^ No cases of thrombosis have been reported.

Physiological hemostasis is a dynamic process, traditionally described in 3 steps. Primary hemostasis leads to platelet aggregation and involves the blood vessel, platelets, and von Willebrand factor (vWF). Secondary hemostasis, or coagulation, starts with the interaction between tissue factor, exposed to the blood stream after endothelial disruption, and FVII, leading to the formation of thrombin (FII), which will activate both branches of the coagulation cascade (extrinsic [triggered by the activation of FVII] and intrinsic [triggered by the activation of FXII]), thus increasing its own production (thrombin burst). Thrombin will eventually be transformed into fibrin, which stabilizes the clot. Tertiary hemostasis, or fibrinolysis, prevents excessive clotting formation through fibrin degradation by plasmin.

Based on the detailed description of a series of 14 French patients, we aimed to (1) characterize clotting abnormalities in mastocytosis, (2) suggest underlying mechanisms for these, and (3) appreciate their relevance in clinical practice.

## METHODS

We used the CEREMAST (*Centre des référence des Mastocytoses*) national database to retrospectively identify patients with mastocytosis and clotting abnormalities. The use of this computer database to store personal information was authorized by the French National Data Protection Commission; CEREMAST authorization: CNIL: No1445939 (October 29, 2010). Overall, 23 regional centers affiliated to the CEREMAST and homogeneously distributed in France were then contacted to obtain more information on potential cases. Patients aged ≥15 years old, with a confirmed diagnosis of mastocytosis (including genetics to determine *CKIT* mutations) according to the World Health Organization (WHO) criteria,^19^ with evidence of a current or past medical history of clinical and/or biological clotting abnormalities were included and analyzed. Patients with the following characteristics were excluded from this study: inherited clotting abnormality, suspicion or confirmation of antibodies interfering with coagulation, vitamin K deficiency, treatments directly interfering with clotting, disease-related or other causes of hepatocellular insufficiency at the time of the study, severe renal impairment (with urea >10 mmol/L). Patients for whom data was deemed insufficient were also excluded. Approval was obtained from the Necker Children's University Hospital (Paris, France) ethical committee. Written informed consent was also obtained from all patients whose data are discussed in this paper in accordance with the declaration of Helsinki.

The clinician from CEREMAST verified, for each patient, the type and classification of mastocytosis according to the WHO criteria^19^ and ensured accuracy of the clinical and biological history. To avoid including patients with pre-existing clotting disorders, a bleeding history and, when available, the results of biological tests performed before mastocytosis onset were collected.

In order to provide a well-recognized and uniform tool, we used the Common Terminology Criteria for Adverse Events (CTCAE), version 4.0, to grade bleeding symptoms. Primary hemostasis was assessed by platelet function assay (PFA) for epinephrine, adenosine diphosphate (ADP), and von Willebrand factor (vWF) antigen (Ag) and activity (Rco). We have excluded classical causes of acquired vWF disease, including hypothyroidism, autoimmune disease, monoclonal gammopathy, aortic stenosis, and solid malignant tumors. Secondary hemostasis was assessed by prothrombin time ratio (PTr), an equivalent of prothrombin time (PT) used in France, expressed in percentage of a reference value (low PTr corresponds to a high PT, normal >70%) as well as by activated partial thromboplastin time (aPTT) ratio (patient's aPTT/reference aPTT in seconds = aPTT ratio or aPTTr, normal ≤1.20), clotting factors levels (F followed by the corresponding roman numeral), and anti-Xa levels. When multiple clotting samples were available, we selected the most abnormal one.

Importantly, note that in view of the retrospective nature of this study, the data available are extremely variable from patient to patient, depending on the degree of detail of clinical records and investigations performed by the clinician who assessed the patient at that time.

For the purpose of clarity, we divided patients into 2 groups depending on whether they predominantly presented with abnormalities of primary hemostasis (group A, #1–4), or secondary hemostasis (group B, #5–14). As discussed later, some patients from group B also had concomitant primary hemostasis abnormalities, but these were not predominant. Tertiary hemostasis was not analyzed due to lack of data.

Literature research was performed using *PubMed* database. Key words included “mast cell”, “mast cell disease,” “mastocytosis,” “clotting,” and “coagulation.” Terms related to specific mast cell mediators and actors of coagulation were also used. Papers were selected from the main results list and from the “related citations” list suggested by PubMed. In addition, we explored the bibliographies of relevant papers.

## RESULTS

From the 21 patients with mastocytosis and clotting abnormalities initially identified via the CEREMAST database, n = 14 met the study's inclusion criteria. Population characteristics are summarized in Tables 1 and 2. Table 1 particularly summarizes each patient's clinical and biological data with regard to their mastocytosis. Clotting findings including clinical presentation, investigations, management, and response to the latter are presented in Table 2  . The symptomatic (mast cell-stabilizing or mediator-targeting drugs) and cytoreductive treatment history, notably the use of steroids, has been clearly mentioned for each patient in Tables 1 and 2.

**TABLE 1:**
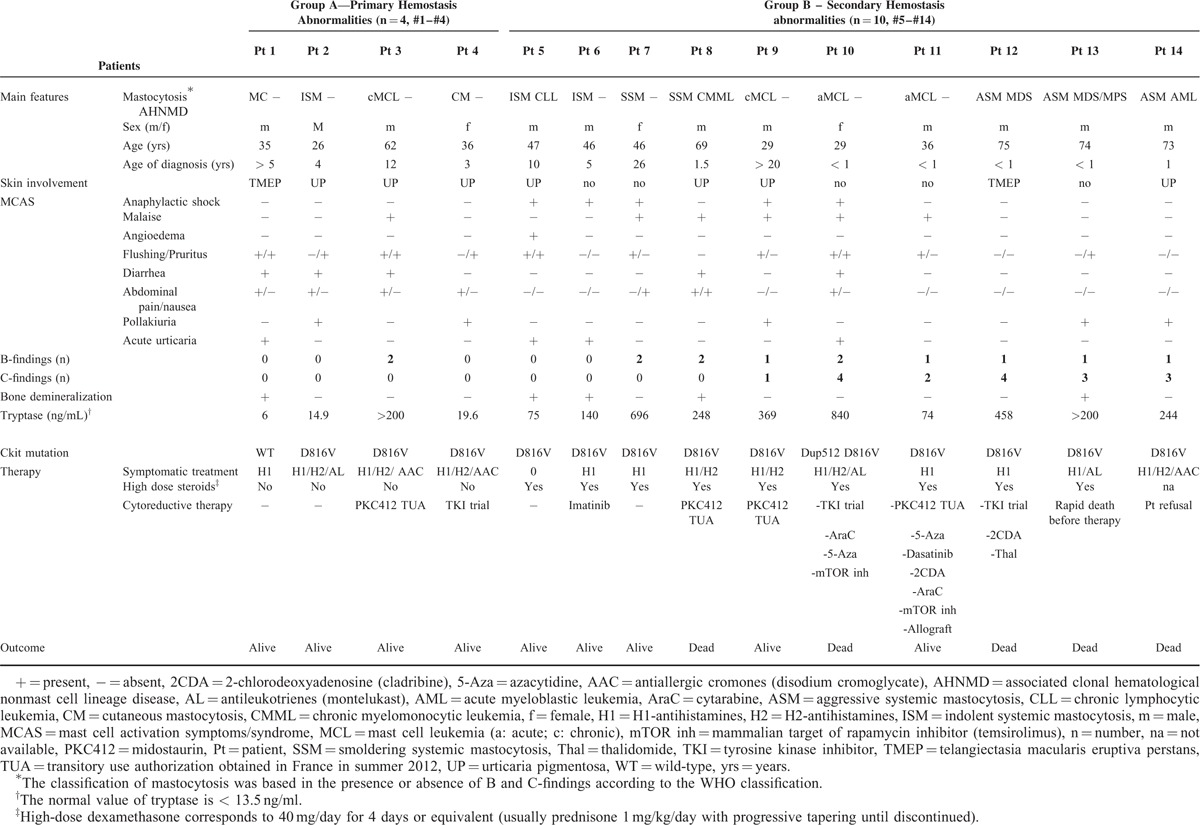
Population Characteristics: Patients Clinical and Laboratory Data With Regard to Their Mastocytosis Type

**TABLE 2:**
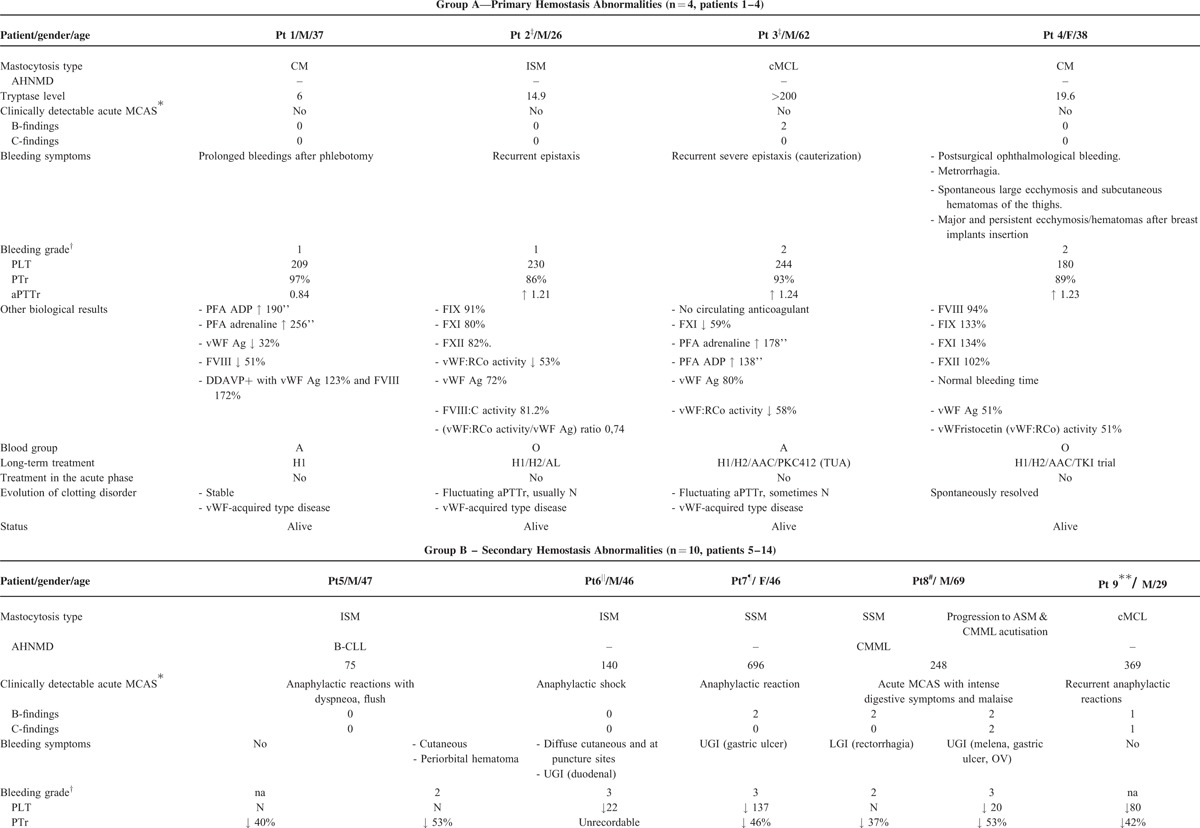
Patients Clinical and Laboratory Data With Regard to Their Bleeding History and Clotting Investigations

**TABLE 2 (Continued):**
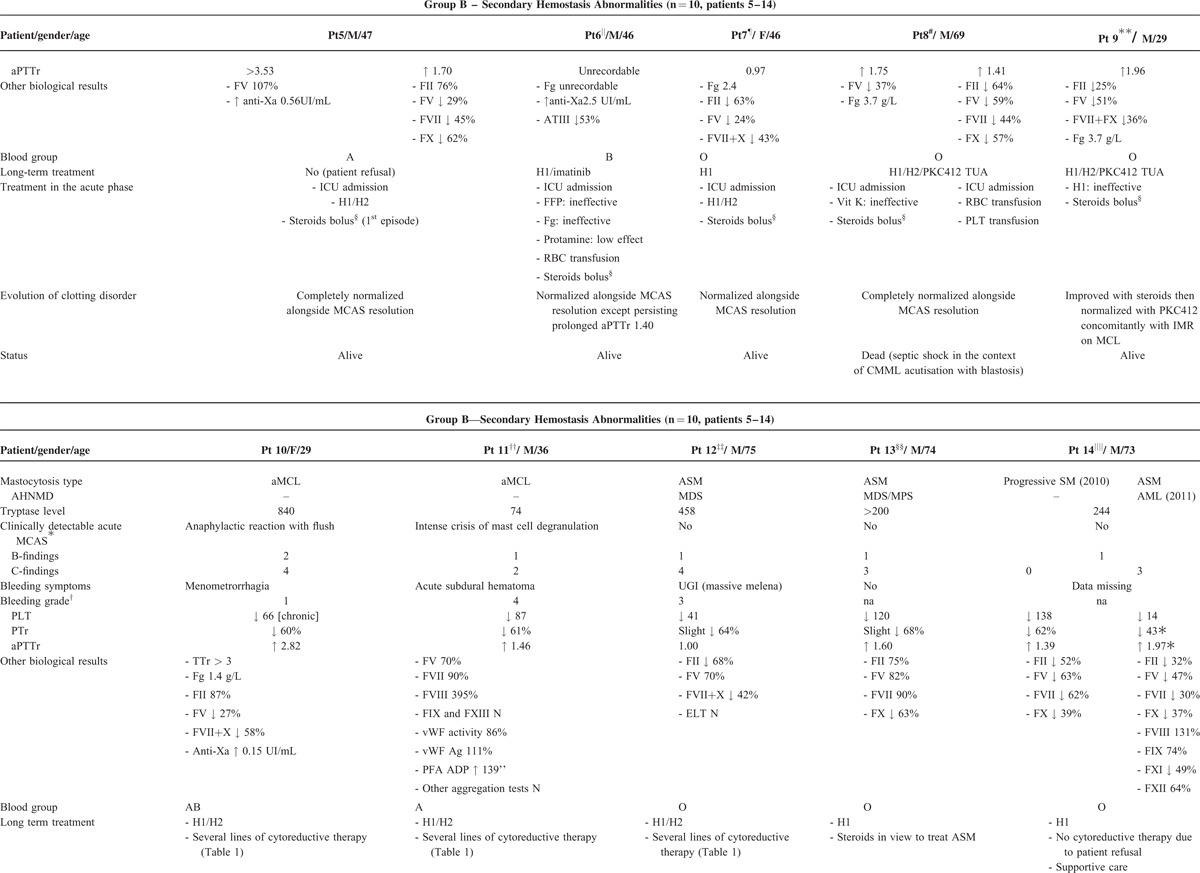
Patients Clinical and Laboratory Data With Regard to Their Bleeding History and Clotting Investigations

**TABLE 2 (Continued):**
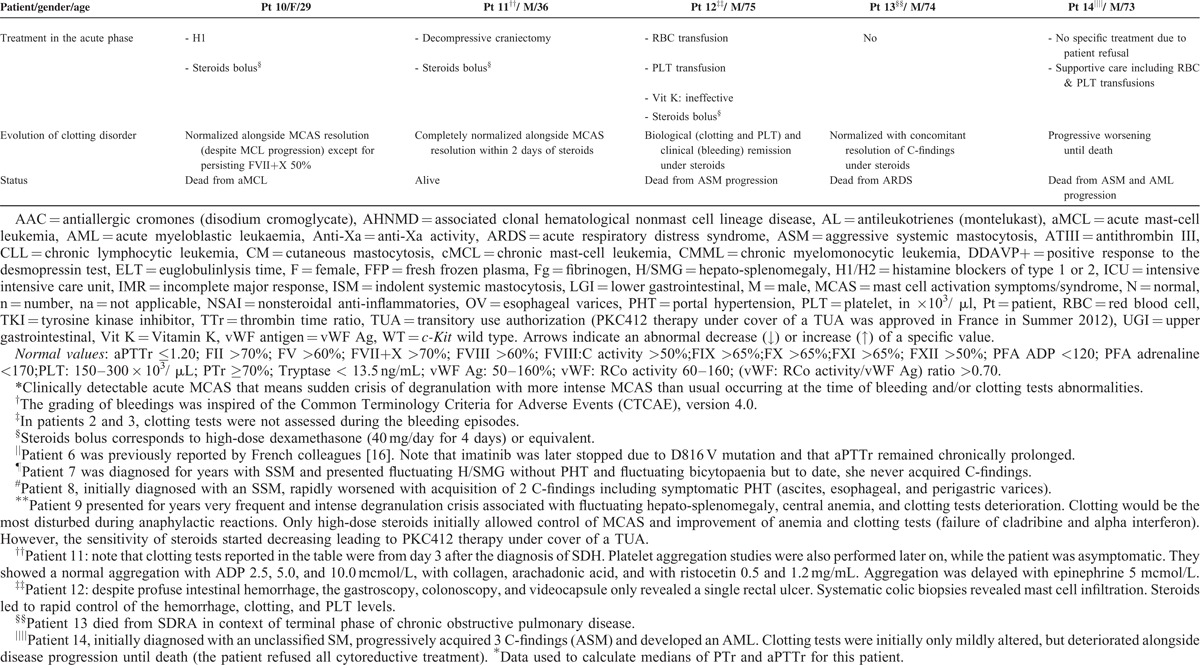
Patients Clinical and Laboratory Data With Regard to Their Bleeding History and Clotting Investigations

Group A (n = 4) is characterized by an impairment of primary hemostasis, a median age of 35.5 (26–62) years, absent (#1) or mild (#2–#4) bleeding symptoms (maximum CTCAE bleeding grade of 2), a majority of indolent mastocytosis (3/4), no C-findings, no AHNMD and no deaths to date. After adjusting reference ranges to the blood group, the diagnosis of vWF disease was confirmed in #1 (type 1 vWF disease: prolonged PFA, proportionate decrease of vWF Ag and FVIII levels), and strongly suspected in #3 (type 2 A or B vWF disease: prolonged PFA, borderline vWF:RCo activity/vWF antigen, normal vWF antigen). Interestingly, #2 and #4 were blood group O, which is typically associated with lower levels of vWF antigen. The clinical presentation of these 2 patients was very suggestive of impairment of primary hemostasis.

**FIGURE 1:**
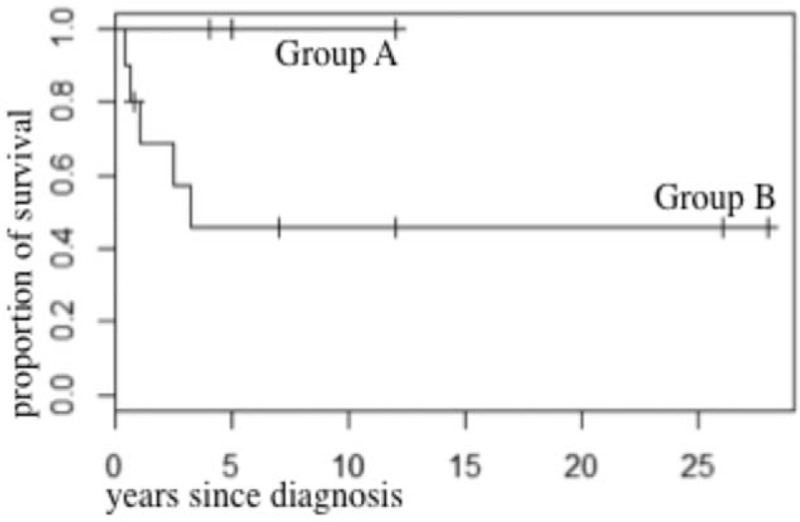
Survival curves in groups A and B (Kaplan–Meier analysis).

In contrast, group B (n = 10) is characterized by an impairment of secondary hemostasis, a median age of 46.5 (29–75) years, and more frequent, more severe, sometimes life-threatening bleeds. Seven out of 9 patients had bleeding symptoms (data missing in n = 1), which were particularly severe (CTCAE grade ≥3) in 5 patients. These included 4 gastrointestinal (#6–#8, #12) and 1 intracranial (#11) bleeds. A majority of patients from group B had an aggressive type of mastocytosis (associated with AHNMD in 5/10) and a significantly reduced survival with 5 deaths among 10 patients and a median survival time of 2.5 years (Fig. 1).

Most of the patients from group B had severe episodes of mast cell degranulation (7/10, #5-#11), and/or high mast cell organ infiltration (8/10, #7–#14) illustrated by B and C-findings with a median of 1.5 C-findings (0–4). Importantly, a median PTr of 53% (out of range [low]-64) and a median aPTTr of 1.75 (1.03-out of range [high]) were recorded in patients with MCAS (n = 7, #5–#11), which suggests a more severe hemostasis impairment profile than that of patients without MCAS (n = 3, #12–#14) who exhibited a median PTr of 64% (43–68) and a median aPTTr of 1.39 (1.00–1.97).

**FIGURE 2:**
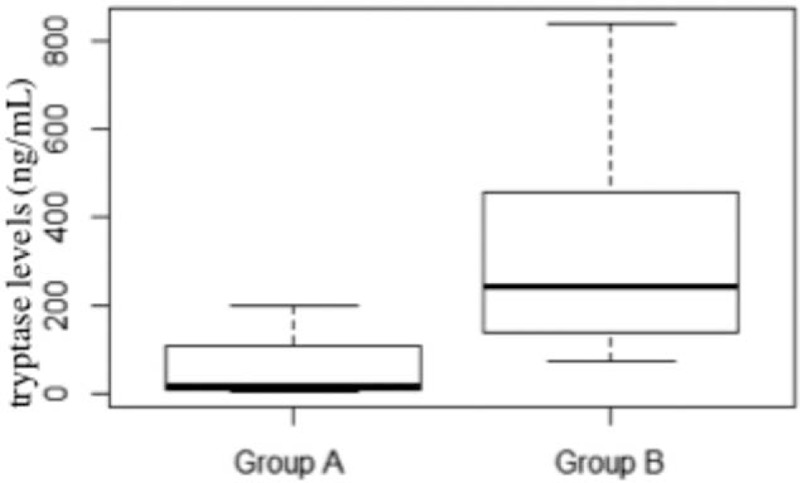
Comparison of tryptase levels (ng/mL) in groups A and B (nonparametric Wilcoxon test).

Tryptase levels were significantly higher in group B compared to in group A (*P* = 0.0235, nonparametric Wilcoxon test, Fig. 2). Moreover, the prevalence of CM/ISM, SSM, ASM/SM-AHNMD, and MCL was, respectively, 75% (3/4), 0%, 0%, 25% (1/4) in group A versus 10% (1/10), 10% (1/10), 50% (5/10), 30% (3/10) in group B where an increased mortality rate was recorded (n = 5, 36% of the overall population, Fig. 1).

Classical management of bleeding including vitamin K infusion, platelet transfusions, and fresh frozen plasma appeared ineffective to achieve hemostasis (#6, #8, #12). By contrast, clotting tests and bleeding symptoms dramatically improved and/or normalized alongside subsidence of the degranulation crisis. The latter either occurred spontaneously (#4) or by targeting blockade of mast cell activation with agents such as antihistamines and, more effectively, high dose of steroids (#5–#13). Failure to control mast cell hyperactivity and aggressive proliferation (C-findings) led to exponential deterioration of coagulation tests and death from the disease (#14).

## DISCUSSION

This study is the first detailed description of several types of clotting abnormalities associated with mastocytosis. Although rare, they can be severe and must be promptly recognized to allow appropriate management. The spectrum of mastocytosis-related clotting disorders includes acquired vWF disease, acquired disorders of the coagulation cascade, and probably of fibrinolysis.

Only few previous case reports had already pointed out clotting impairment in the context of mastocytosis.^6–18^ However, the disparity in the population characteristics, the lack of systematic clinical/laboratory data, and of accurate mastocytosis classification mean that a clear hemostatic analysis from those reports remains poor. From our series, the description of our cases and the therapeutic implications of the background disease on the acquired hemostasis disorders shed new light on the significance and mechanisms involved in clotting disorders in the context of mastocytosis.

The mucocutaneous bleeds observed in group A are characteristic of abnormalities in primary hemostasis and reflect the role of von Willebrand factor in platelet adhesion and aggregation. This can be impaired by heparin, a substance present in mast cells, and released during degranulation. Indeed, heparin can bind to vWF, therefore preventing platelet adhesion via GPIb, and platelet aggregation via GPIb and GPIIbIIIa.^20^ This may explain why patients with low baseline vWF levels (either secondary to vWF disease or to blood group O) are more prone to mucocutaneous bleeds during mast cell activation syndromes. Therefore, by checking the blood group and testing for vWF disease in patients with mastocytosis, clinicians could potentially identify those at higher risk of bleeding and provide education on prophylactic measures, as well as the plan for possible complications following invasive procedures (for example, #4 developed extensive mammary hematomas after breast implant).

Impairment of primary hemostasis may coexist and be concealed by abnormalities in the coagulation cascade, particularly in subjects who are blood group O (60% of patients in group B, versus a prevalence of 43% in the normal French population according to the *Institut National de la Transfusion Sanguine* [INTS]). Patients #5 and #6 illustrate this particularly well as they developed hematomas/conjunctival bleeds, which are characteristic of primary hemostasis abnormalities.

The abnormalities of secondary hemostasis observed in group B were more pronounced in acute episodes of mast cell degranulation: patients with MCAS with or without organ infiltration (ie B and/or C-findings) had more bleeding symptoms than those where no clinically detectable MCAS was reported (Table 2  ). Bleeds in group B tended to be internal and therefore characteristic from abnormalities of the coagulation cascade (as opposed to mucocutaneous bleeds, which are often related to abnormalities in primary hemostasis). They were typically severe and life-threatening (4 gastrointestinal [#6–#8, #12] and 1 intracranial [#11]). The major role of MCAS exacerbations is further demonstrated by the rapid resolution of bleeding symptoms and/or clotting tests abnormalities as the MCAS subsides, even in cases where they were initially very severe.

This has major therapeutic implications in clinical practice and explains why standard hemostatic management with vitamin K, fresh frozen plasma, or platelet transfusion is insufficient. Indeed, to overcome mast cell hyperactivity and subsequent uncontrolled degranulation leading to an anticoagulant state, it is crucial that mast cell mediator blockers and mast cell stabilizers are used. We suggest the use of steroids in the acute setting and of maintenance treatment in the chronic setting for patients who have persistent symptoms of degranulation.

Regarding the role of mast cell mediators released during degranulation, tryptase, heparin, and antithrombin III are the 3 main molecules interfering with standard coagulation tests. The prolonged aPTT observed in our patients could result from several intricate mechanisms: the activating effect of tryptase on fibrinogenolysis^21–25^ and on kininogenolysis,^26,27^ the overall anticoagulant role of heparin through upregulation of thrombomodulin, which activates protein C and S pathways,^28,29^ and the release of antithrombin III.^30,31^ Interestingly, all these mechanisms can only account for prolonged PT secondary to an anticoagulant activity of the common pathway, as there is no known anticoagulant interaction between mast cell mediators and the extrinsic pathway. Therefore, we should expect PT to be prolonged only in the presence of prolonged aPTT, which is not the case (#7, #12), suggesting that other unknown mechanisms are involved.

Finally, the overrepresentation of aggressive mastocytosis types, increased mortality rate and higher tryptase levels among patients with abnormalities of secondary hemostasis raises a question about the role of standard clotting tests in mastocytosis. Although the disease by itself, and particularly AHNMD, can impair coagulation, this seems unlikely here given the close relationship between clotting, episodes of degranulation, and response to steroids. As a consequence, and pending further research, targeted coagulation history and standard blood tests (PT, aPTT) might become relevant markers of poor prognosis in this population, as they would reflect the increasingly uncontrolled clonal activation and proliferation of mast cells.

The main limitations of this study reside in its retrospective analysis, the small number of cases, the delays between the onset of symptoms and blood testing, the lack of systematic platelet aggregation tests, and the intrinsic limitations of coagulation tests (a constantly evolving process). The overall clinical picture and transient character suggest nevertheless that these abnormalities are related to mast cell activity rather than to a pre-existing factor deficiency. We are unable, at this stage, to fully understand the disparity between clinical and laboratory pictures (which could be partly related to under-diagnosis and to the above limitations).

## CONCLUSION

This study suggests that mastocytosis can be a cause of acquired clotting abnormalities, particularly during mast cell activation and degranulation. We suggest that these patients are tested for blood group O and vWF disease as these increase the risk of mucocutaneous bleed during mast cell degranulation. Severe, potentially life-threatening bleeds were described in half of the patients with impairment of the coagulation cascade. Mast cell disease-controlling drugs, particularly steroids, were the only effective way to achieve hemostasis. A larger and prospective study systematically assessing clotting in patients with mastocytosis would be required to confirm whether in the future, standard or specific clotting tests could be used as a marker of mast cell activity and potentially of poor disease prognosis.
